# Investigation of DNA Damage and Cell-Cycle Distribution in Human Peripheral Blood Lymphocytes under Exposure to High Doses of Proton Radiotherapy

**DOI:** 10.3390/biology10020111

**Published:** 2021-02-03

**Authors:** Justyna Miszczyk

**Affiliations:** Department of Experimental Physics of Complex Systems, Institute of Nuclear Physics Polish Academy of Sciences, 31-342 Krakow, Poland; Justyna.Miszczyk@ifj.edu.pl; Tel.: +48-126628409

**Keywords:** human peripheral blood lymphocytes (HPBLs), proton radiotherapy, premature chromosome condensation (PCC) test, X-rays, cell-cycle, DNA damage, high doses, excess fragments, rings

## Abstract

**Simple Summary:**

Radiotherapy is a cornerstone care therapy for many tumors. Despite permanent advances in ra-diation dose delivery, there are unmet needs for further improvement. In principle, proton therapy offers a substantial clinical advantage over conventional modalities using photons in uniform-dose delivery of radiation to tumors, along with significant reductions in the harmful effects on normal tissue. However, the effect and mechanisms of a single high-dose delivery remain unclear. The study aimed to systematically observe and compare the biological effects of DNA damage and cell-cycle phase distribution in the human peripheral blood lymphocytes ex vivo irradiation model of normal tissue after proton versus conventional radiotherapy (X-rays). The effects induced at a single high-dose radiation exposure at a dose range of 8.00–20.00 Gy were studied. The results in-dicate a different distribution of DNA damage following high doses of irradiation with protons versus photons between donors, types of radiation, and doses. The results illuminate the cellular and molecular mechanisms that underlie differences in the distribution of DNA damage and cell-cycle phases. An understanding of the mechanisms in the distinct pathways induced by radiation can facilitate the development of more efficient radiotherapies with beneficial immunological conse-quences.

**Abstract:**

This study systematically investigates how a single high-dose therapeutic proton beam versus X-rays influences cell-cycle phase distribution and DNA damage in human peripheral blood lymphocytes (HPBLs). Blood samples from ten volunteers (both male and female) were irradiated with doses of 8.00, 13.64, 15.00, and 20.00 Gy of 250 kV X-rays or 60 MeV protons. The dose–effect relations were calculated and distributed by plotting the frequencies of DNA damage of excess Premature Chromosome Condensation (PCC) fragments and rings in the G2/M phase, obtained via chemical induction with calyculin A. The Papworth’s *u* test was used to evaluate the distribution of DNA damage. The study shows that high doses of protons induce HPBL DNA damage in the G2/M phase differently than X-rays do. The results indicate a different distribution of DNA damage following high doses of irradiation with protons versus photons between donors, types of radiation, and doses. The proliferation index confirms the impact of high doses of mitosis and the influence of radiotherapy type on the different HPBL response. The results illuminate the cellular and molecular mechanisms that underlie differences in the distribution of DNA damage and cell-cycle phases; these findings may yield an improvement in the efficacy of the radiotherapies used.

## 1. Introduction

Radiation therapy (RT), also called radiotherapy, is a cornerstone care therapy for many tumors. It uses various radiation doses and treatment schemes to kill cancer cells and shrink tumors. Despite permanent advances in high-precision dose delivery [1], there are unmet experimental and clinical needs for ex vivo biological validations and further improvement [2]. In principle, proton therapy (PT) offers a substantial clinical advantage over conventional modalities using photons or electrons [2]. The superior and unique depth–dose distribution properties of protons at the site called the “Bragg Peak” are used to achieve higher and more uniform dose delivery of radiation to tumors, along with significant reductions in the harmful effects on normal tissue [2,3]. This improving local control of tumors is simultaneously reducing toxicity and improving future quality of life [3]. Meanwhile, recent studies suggest that FLASH radiotherapy (FLASH RT)—in which radiation is delivered at ultra-high dose rates—reduces toxicity in normal tissues while effectively killing tumor cells [4,5]. Data based on preclinical studies have suggested that RT, especially with higher single doses of 20–25 Gy, can substantially stimulate anti-tumor T-cell immunity and increase the T-cell response to help control tumor growth [6]. Thus, in recent years, it has been suggested that a single high dose of radiation may be also used in radiotherapy [7]. However, the biology and mechanisms underlying FLASH RT, as well as the effect of a single high-dose delivery, remain unclear [5].

In a previous series of studies, we demonstrated that therapeutic proton irradiation and X-rays induce DNA damage and cell death in normal tissue by various modes at doses in the range of 0.3–4.0 Gy [8,9,10]. These differences influence the type, incidence, and intensity of the adverse effects of radiation therapy while allowing the development of predictive biomarkers of clinical outcomes and facilitating the selection and/or stratification of patients or patient cohorts for a given modality of radiation treatment [8,9,10,11].

Human peripheral blood lymphocytes (HPBLs) are arrested predominantly at a DNA presynthetic stage in the cell cycle; only CD3+ T cells enter the cell cycle by stimulation with phytohemagglutinin (PHA) [12]. They circulate throughout the body and represent normal tissue [8,13]. Contradictory roles have been described, although many have postulated that they may be critical targets for radiotherapy and/or immunotherapy [13,14]. Conventional biological methodologies for biodosimetry as a dicentric assay for doses up to 5 Gy have limitations regarding cell death, delays in the cell cycle, and a gradual reduction of cell numbers reaching mitosis [15]. The chemically induced premature chromosome condensation (PCC) method—using chemical inhibitors of protein phosphatases (i.e., calyculin A)—overcomes the major problems of the radio-induced cell cycle arrest at high doses [16,17]. Calyculin A or okadaic acid are inhibiting serine/threonine phosphatases type 1 and type 2A, thus leading to the induction of PCC in any phase of the cell cycle [15,16].

The study aims to systematically observe and compare the biological effects of DNA damage and cell-cycle phase distribution in the HPBLs ex vivo irradiation model of normal tissue at a single high-dose radiation exposure after proton versus conventional radiotherapy (X-rays). The drug-induced PCC methodology was proposed to study the effects induced at a dose range of 8.00–20.00 Gy.

## 2. Results

### 2.1. High Doses of Protons Induce HPBLs DNA Damage in the G2/M Phase Differently Than X-rays

**Figure 1:**
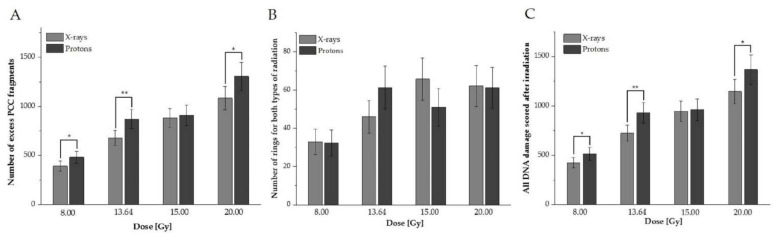
Average values for human peripheral blood lymphocytes (HPBLs) irradiated with doses of 8.00, 13.64, 15.00, or 20.00 Gy with protons vs. X-rays (**A**: excess fragments, **B**: rings, **C**: all damage). The error bars represent the standard deviation. *, *p* < 0.05; **, *p* < 0.01.

To investigate cell-cycle distribution and DNA damage in human peripheral blood lymphocytes under exposure to various high doses of proton radiotherapy, reference X-ray photons were used. DNA damage was measured as the excess fragments and rings of chromosomes in a total of 5000 G2/M phase cells. The mean value of excess fragments for all studied donors was 29.8 ± 8.11 (with no rings detected) in non-irradiated cells. Figure 1A–C presents a comparison between studied types of radiation for excess fragments, rings, and all DNA damage, respectively, as an average number of induced damages for 10 donors after 8.00, 13.64, 15.00, or 20.00 Gy.

For all doses except 15.00 Gy, a statistically significant higher DNA damage value scored as excess PCC fragments after proton radiotherapy was observed (Figure 1A). Their frequency for both protons and X-rays increased with the dose. For rings (see Figure 1B), did not observe statistically significant differences between the studied types of radiotherapy. Their frequency was not related to the dose and type of radiation (Figure 1B). To study the impact of rings and excess fragments, all observed DNA damage was summarized (Figure 1C). The results confirmed that a significantly higher average total value of DNA damage was observed after protons, except with the 15 Gy dose. These results indicate that high doses of 60 MeV protons can induce HPBL responses (presented as a higher frequency of DNA damage, on average, among donors) differently than conventionally used photon radiotherapy. On average, high doses of radiation can induce different types of damage, which are correlated with an increased dose for excess fragments, whilst for rings, data were not related to types of radiation and dose.

### 2.2. The Distribution of DNA Damage Following High Doses of Irradiation with Protons Versus Photons in HPBLs Differs between Donors, Types of Radiation, and Doses

**Figure 2:**
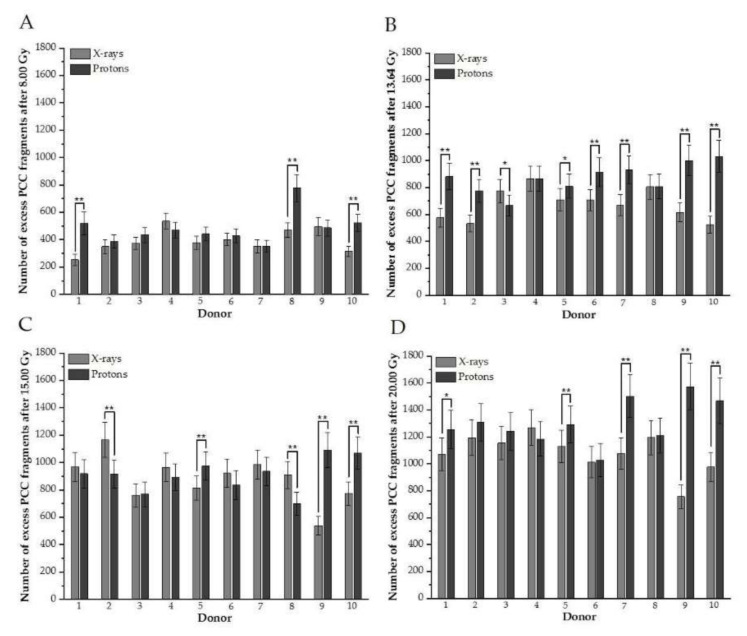
(**A**–**D**). Distributions of the number of induced excess fragments in G2/M cells irradiated with protons vs. X-rays of 8.00, 13.64, 15.00, and 20.00 Gy, respectively, for each donor. Each column represents scored excess fragments with error bars as a standard deviation. *, *p* < 0.05; **, *p* < 0.01. Donor #1, #2, #7, #9, and #10 are female; #3, #4, #5, #6, and #8 are male donors.

**Figure 3:**
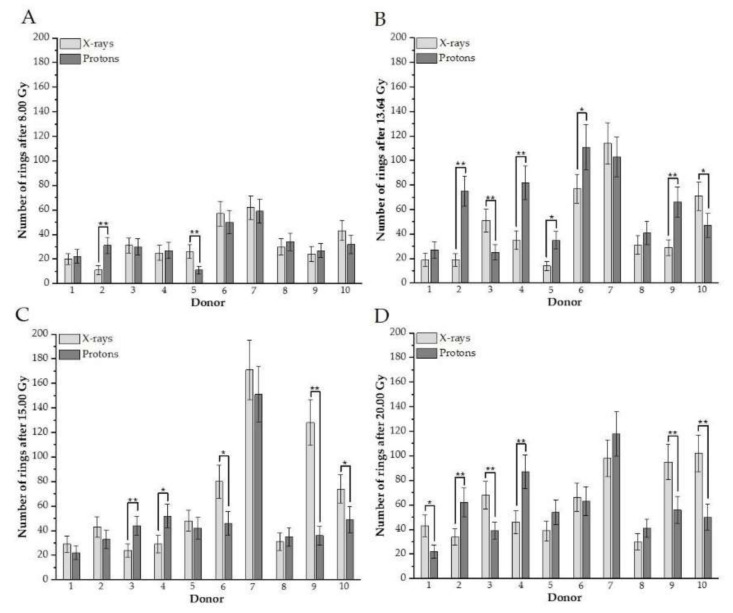
(**A**–**D**). An individual number of rings between donors in G2/M cells irradiated with different doses of protons vs. X-rays (**A**: after 8 Gy, **B**: 13.64, **C**: 15.00 Gy, **D**: 20.0 Gy). Each column represents scored rings with error bars as a standard deviation. *, *p* < 0.05; **, *p* < 0.001. Donor #1, #2, #7, #9, and #10 are female; #3, #4, #5, #6, and #8 are male donors.

To examine the contribution of DNA damage in each donor to the mean value presented in Figure 1, plots for every studied dose and donor, comparing excess fragments after protons versus photons, were created (Figure 2A–D). The analogous plot was prepared for rings (Figure 3A–D).

The results confirm individual differences among donors in excess fragments. After 8 Gy for three donors (#1, #8, and #10), a higher frequency of excess fragments was observed for protons (Figure 2A), showing that these three donors mostly contributed average values of excess fragments obtained for HPBLs, as presented in Figure 1A. For nearly all donors after 13.64 Gy (except #4 and #8), statistically greater DNA damage—presenting as excess fragments—was observed after proton treatment (Figure 2B). Similar results were observed after 20 Gy (Figure 2D). Interestingly, for the 15-Gy dose of X-rays for six donors (#1, #2, #4, #6, #7, and #8), a higher frequency of PCC fragments was observed after X irradiation (two were statistically significant) (Figure 2C). For X-rays and each dose of 8.00, 13.64, 15.00, and 20 Gy of protons for a particular donor, the number of rings is shown in Figure 3A–D, respectively. Similarly, as with excess fragments, the results confirm individual differences among individuals without a general tendency for this type of DNA damage.

One of the primary interests with photon and particle therapy is DNA damage-distribution studies to evaluate dose heterogeneity. Table 1 shows Yield ± S.D., the dispersion index (DI), and *u* values calculated to test whether the distribution of aberrations follows a Poisson for all donors in lymphocytes exposed to various doses of X-rays and protons. The distribution of extra fragments and rings is overdispersed (with *u* values > 1.96) with all doses and types of radiation (see Table 1). Notably, after X-rays, a lower over-dispersion was observed for both excess fragments and rings. The highest over-dispersion was observed for excess fragments irradiated with protons. 

**Figure 4:**
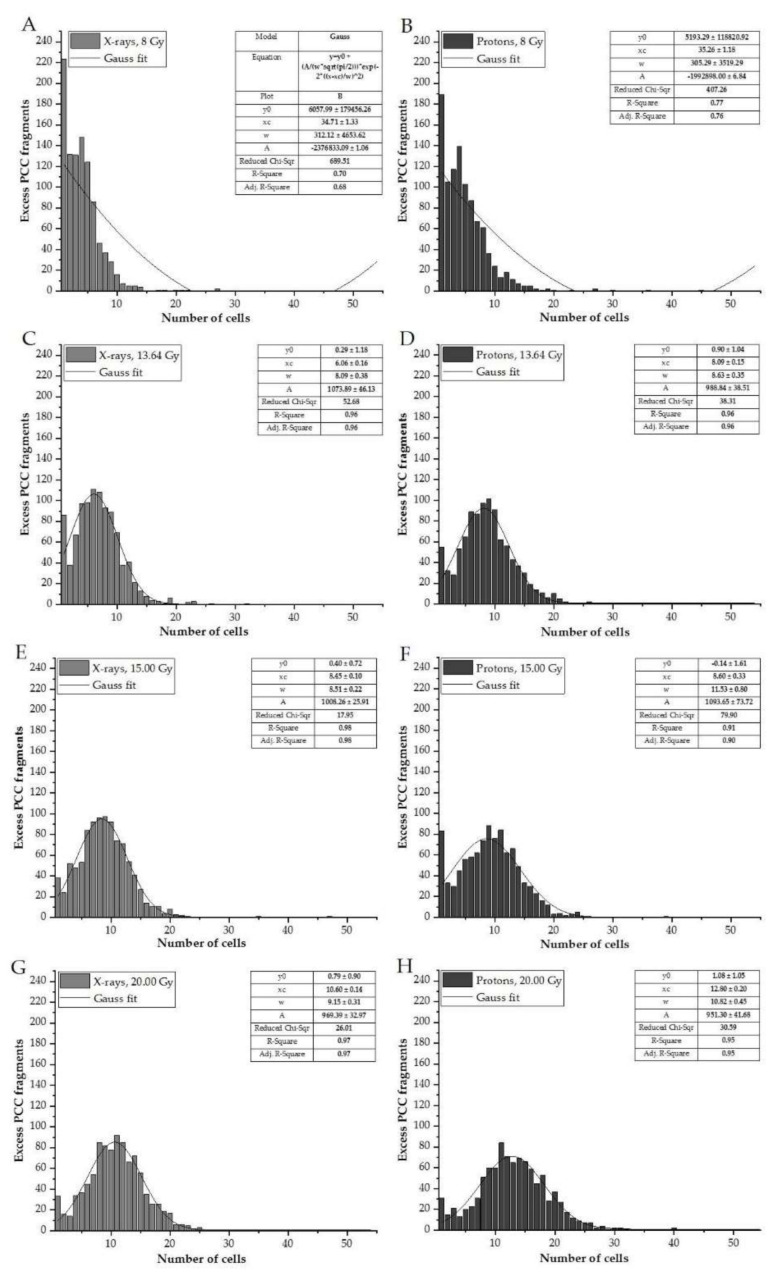
Frequency distribution of the number of excess fragments in cells irradiated with various doses of X-rays (**A**,**C**,**E**,**G**), compared to those treated with protons (**B**,**D**,**F**,**H**). The parameters determined for Gaussian fitting are presented in the tables located with each histogram.

The frequency distributions (histograms) of the measured excess fragments are calculated for the HPBLs of all donors irradiated with various doses of protons (presented in Figure 4B,D,F,H) and X-rays (Figure 4A,C,E,G). For each dose, data were stratified in ranges presenting the number of G2/M cells carrying 0–54 excess fragments. To determine whether the distribution of the number of excess fragments in cells follows a normal distribution, Gauss fitting was performed. Gaussian functions appear in many contexts in the biological and medical sciences. The fit parameters are presented in the tables connected to each histogram.

The frequency distribution of the observed number of excess fragments differs according to the doses and type of radiation, indicating a more scattered distribution for protons compared to X-rays (Figure 4F,H: the presence of more than 20 cells with excess fragments compared to Figure 4G). The resulting R-squared values show a very high goodness-of-fit for DNA damage obtained in the dose range 13.64–20.00 Gy (R^2^ of 0.91–0.98, irrespective of the radiation type used). Lower R^2^ values (0.70 and 0.77) were observed with the 8.00-Gy dose. 

### 2.3. The Proliferation Index Confirms the Impact of High Doses on Mitosis and the Influence of Radiotherapy Type on the Different Normal Tissue Responses

The cell-cycle stage distribution was systematically investigated after stimulation with PHA in a PCC assay. Table 2 presents the percentages of cells classified into the five cell stages (G1, S, G2, M, A, and nucleated), scored for the first 200 cells after irradiation with 8.00, 15.00, 13.64, and 20.00 Gy of X-rays and protons. Furthermore, the cell-cycle distribution was measured at the starting time of irradiation among controls (0.00 Gy) for all donors, achieving the following percentages, on average: G1 (1.20%), S (2.10%), G2 (2.85%), M (0.70%), A (0.65%), and nucleated (92.5%).

**Figure 5:**
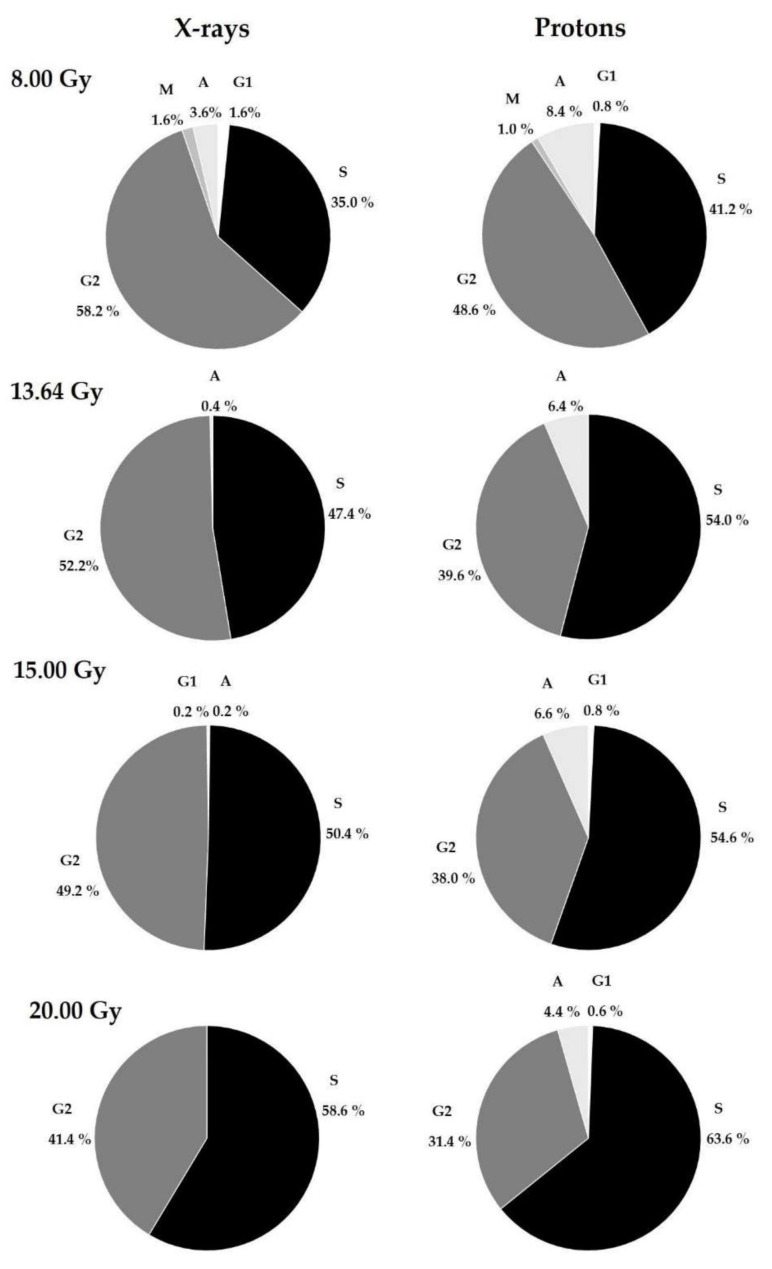
Percentages of cells in the G1, S, G2, M, and A phases at each dose for 10 donors after X-rays and protons in PHA-stimulated HPBLs during exposure at various doses: 8.00, 13.64, 15.00, and 20.00 Gy.

These results indicate that as the dose increases, the number of nucleated cells for both studied types of radiation increases to comparable values (≅97%). The remaining 3% are represented mostly by the S and G2 phases (see Table 1). Due to the presence of numerous non-divided cells, a more detailed analysis was performed by scoring an additional 50 cells in the G1, S, G2, M, and A phases to determine their distribution into various cell-stages, considering the dose and type of radiation. The results are presented in Figure 5. The cell-cycle distribution among controls (0.00 Gy) for all donors reached the following percentages, on average: G1 (13.0%), S (25.4%), G2 (33.6%), M (17.0%), A (11.0%).

The results confirm that the largest proportion of lymphocytes were in the S and G2 phases after irradiation at high doses. For both types of radiation, as the dose increased, the number of S-phase cells also increased and was higher for each dose after proton radiotherapy (Figure 5). Interestingly, the reverse effect was observed for G2-phase cells. As seen in Figure 5, for both types of radiation, the percentage of G2-phase cells gradually decreased, reaching higher values after X-ray treatment. Cells in the A-phase were also observed, with a higher proportion after proton irradiation. As with S-phase cells, A-phase cells gradually decreased for both radiation types with increasing doses. M-phase cells were observed only after treating HPBLs with 8.00 Gy of photons or X-rays. These results indicate that the presence of G1-phase cells is not dependent on the type of radiation or the dose.

## 3. Discussion

The purpose of radiation therapy is to maximize tumor cell killing with no or minimal normal tissue complications [1,2]. Spectacular technological developments over the last decade, along with the establishment of new proton and ion centers around the world, are likely to increase the number of patients treated with protons for certain tumor types, including pediatric tumors [2]. However, despite the recent promising studies and clinical translation of FLASH radiotherapy and single high doses [18], greater efforts are urgently needed to study the effects of high doses on normal tissue responses.

In the following section, the effects of a single fraction of high-dose radiation on DNA damage and the cell cycle in HPBLs, representing the immune system and a normal tissue model are discussed. The PCC method was applied as a reliable tool for assessing radiation-induced damage and cell-cycle perturbations after high doses of irradiation [16,17]. The assessment of high-radiation doses by biological methods is difficult, due to the inhibitory effect of radiation on cell proliferation [19]. The PCC method provides insights into the mechanisms of chromosome condensation, and the fact that PCC can be induced in cells within minutes after irradiation was recognized as a valuable tool for investigations in chromosome dynamics and kinetics [20]. Furthermore, the relevance of this technique to DNA damage assessment has been confirmed in the dose range of 5–25 Gy for various types of radiations [21]. G2/M PCC is typically used to score structural chromosomal aberrations after radiation exposure; thus, we focused on this cell-cycle stage to score excess PCC fragments and rings [17,21].

The presented study indicates that high doses of protons induce DNA damage in the G2/M phase differently than X-rays do in an equal dose. On average, the greater DNA damage (scored as excess PCC fragments) was observed after proton therapy. Regardless of the radiation type, the frequency of excess fragments showed a clear increase with the dose. These findings align with previous studies, which have reported that 60 MeV protons at doses above 1.75 Gy were more effective in producing DNA damage than 250 kV X-rays in HPBLs [8,17,22]. Excess acentric fragments result from incomplete repairs. Several other studies have reported that protons inflict more double-stranded breaks (DSBs) and more complex DNA than photon irradiation, in both normal and tumor cell lines [23,24,25]. Clustered DNA lesions are the consequence of the difference in radiation quality; these may be more difficult to repair [24]. Thus, for clinical treatment planning, a constant RBE value of 1.1 is used to calculate the equivalent biological dose for the reference dose of gamma photons generated by a Co-60 source [24,25]. Observations may also be explained by studies from Oeck S. et al. [26]. Studying murine prostate cancer cells (TrC1) and murine embryonal fibroblasts (MEF), these researchers observed differences suggesting that Bragg-peak protons at a dose of 3 Gy can induce several DNA lesions in a restricted area, potentially resulting in DNA lesions with greater complexity than those induced by X-ray photons [26]. An intriguing result from these studies, at first glance, is that there are no differences in DNA damage after a dose of 15 Gy. This may be explained by the possibility of observed DNA damage saturation after 15 Gy or 20 Gy [27]; therefore, this highlights the necessity of using more donors in future studies.

Kanda R. et al. proposed the scoring of rings for biological dosimetry purposes in chemically induced, prematurely condensed chromosomes after the lymphocytes are cultured [27]. Among the chromosomal aberrations recorded in these studies, chromosome rings showed the lowest frequency in G2/M PCC cells, without dependency on dose and type of radiation. A comparison with the literature is difficult because most publications present dicentrics, translocation, or excess PCC fragments distributions, while few present PCC rings [28]. Generally, the frequency of PCC rings has been reported to increase at doses up to 20 Gy, indicating their suitability for dose assessment after high-dose radiation exposures [29]. For low-LET (Linear Energy Transfer) radiation, the few reported dose–effect curves show remarkable differences in their coefficients, as well as in the distribution of rings among cells [21,29]. Nevertheless, the presented studies align with previous research, which has reported a similar extra fragments/rings ratio—10 times higher for acentric fragments [21]. Notably, the calyculin A concentration, hypotonic treatment, and slide-preparation process could also have influenced results [30]. Although the scoring criteria were carefully applied, the morphology of rings is sometimes extremely difficult to distinguish from the small chromosomal or chromatid fragments [31]. Despite considerable progress in recent years, the precise mechanisms involved in the formation of rings versus acentric fragments remain a long-standing controversy in radiation cytogenetics. Ring chromosomes are usually formed as a result of two double-stranded DNA breaks from two terminal breaks in both chromosome arms, followed by linking of the broken ends; alternatively, ring chromosomes are formed from the joining of one broken chromosome end with the opposite telomere region [32]. The presence of rings causes mitotic instability, leading to cell death. Additional experimental data focused on the mechanism of ring formation could clarify the differences in radiation-induced frequencies reported using low- and high-LET radiation.

The obtained results show a strong inter-individual difference in DNA damage exposed to both types of radiation (Figure 2 and Figure 3). The extent of the effect does depend on the dose, donor, and type of DNA damage. These differences in the amount of DNA damage in HPBLs have been seen in several studies comparing healthy donors and cancer patients [8,19]. The same received dose may result in dissimilar cytotoxic and genotoxic effects. Observed differences in response to various types of radiation may result from many patient factors [33]. The genetic component is significant for individual radiosensitivity; however, environmental factors, lifestyle, diet, physical activity, and hormonal status also influence the observed effects [8,34]. It has been observed that the proportion of people who exhibit sensitivity to irradiation increases with the dose received, although the correlation is not directly proportional [34]. These observations are important in terms of different responses of patients after the same dose is delivered to the tumors. Further studies should use adequate markers to investigate the role of specific repair pathways that may explain inter-individual differences. Additionally, a correlation study between age, sex, and other confounding factors will help to understand observed effects, but the actual study group is small for such detailed analysis.

When the cell distribution of extra fragments was considered, a clear over-dispersion was observed. Interestingly, a lower over-dispersion was apparent after X-ray treatment for both excess fragments and rings. The highest over-dispersion was noted for excess fragments after proton exposure. A similar variation in over-dispersion has also been reported in other works [29,31,35]. This may stem from the difference in radiation quality. When irradiation is homogeneous, a Poisson distribution of aberrations in cells is expected. It is known that proton beams produce dense ionization tracks due to spatially localized energy deposition in “sequential bursts”, particularly at higher doses [8,23,36]. Using the comet assay, our recent study demonstrated that proton therapy was more effective at high radiation doses [22]. Consequently, the kinetics of repair of DBSs induced by the proton beam versus X-rays in lymphocytes were different [22]. Moreover, non-Poisson distributions are frequently reported using the PCC procedure without Colcemid treatment, as applied in this study [27].

In this study, the frequency distribution (histograms, Figure 4) of the number of excess fragments was examined. There is a lack of understanding of the DNA damage distribution induced by proton beam therapy; therefore, the cellular DNA damage response and repair pathways are not fully understood [23]. For each dose, data were stratified for ranges presenting the number of G2/M cells carrying 0–54 excess fragments. The observed distributions with proton radiotherapy are more scattered when compared to those resulting from X-ray treatment, confirming that DNA damage induced by proton irradiation is affected by the different track structure of the energy deposition. Interestingly, the number of excess fragments per cell resembles the Gaussian distribution, regardless of the radiation type used. Uniform dose deposition of low-LET radiations (i.e., X-rays or γ) results in a random damage distribution, well-described by Poisson statistics [36,37]. Protons are usually considered low-LET radiation [36]. Results may be explained by observations from by Distela L.V.R. et al. [37]. They found that a normal distribution fits well with chromosomal aberrations scored in cells of healthy donors and non-exposed cancer patients, using a three-color FISH analysis [37]. It is possible that the cells with the most extensive DNA damage had undergone cell death before the presence of the DNA damage. In our previous study, we determined the differences between proton and X-ray irradiation in cell viability and cell death within the first few hours following irradiation in the dose range 0.3-4.0 Gy [9]. The number of viable cells at a 4.0 Gy significantly decrease to 46.1 ± 2.7% for protons. Using a higher dose range of 8.00–20.00 might be essential for studying cell death, but it is difficult using HPBLs. More systematic studies are needed to investigate DNA damage distribution between photon and proton/ions in different populations (healthy, cancer, exposed) to identify the greatest risks and adverse effects of radiotherapy.

The responses to DNA damage caused by irradiation are visible as cell cycle alterations. The obtained values for non-irradiated cells corroborate the findings of previous work on HPBLs [12]. Results confirm that the number of nucleated cells for both types of radiation increase with the dose. The data indicate that radiation exposure causes a dose-dependent increase in nucleated and S cells, coincident with a decrease in the G2/M-PCC index. The presence of cells primarily in the S and G2 phases of the cell cycle was observed in the PCC assay, reflecting the cell-cycle arrest induced by ionizing radiation. As seen in Figure 5, the M cells were observed only after 8.00 Gy. This implicates possibility for analyzing chromosome aberrations only in the G2 phase. It is known that DSBs caused by ionizing radiation induce G1/S checkpoint arrest; if this is not maintained, cells are allowed to enter the S phase [38,39,40]. The presence of DNA damage and chromosome breaks activates the G2/M checkpoint, thereby rapidly arresting the cell cycle in G2 [41]. Rodriguez K.F. et al. report that cells with incomplete chromosome elements and incomplete chromosome exchanges are selectively blocked at G2 after radiation [42]. Therefore, the frequency of cells blocked between G1/S and G2 accumulation may yield an increase in G2-PCC cells after radiation in the HPBL ex vivo radiation model. Furthermore, by focusing on the contrastive change of irradiated HPBL cells in the S, G2/M, and A phases, presented studies showed that the cell cycle index is dependent on doses and type of radiation. Analyzing cells in particular stages shows that protons arrested more than X-rays cells in the S phase. Radiation also lowers the number of cells in the G1 phase. Interestingly, the proportion of A-stage cells decreases in a relatively constant manner with proton treatment, whereas this was not observed after 20 Gy of X-rays. This observation is corroborated by other experiments, which showed that heavily damaged cells require longer periods to pass the G2-phase of the cell cycle than lightly damaged cells [35,40,43]. The extent of the observed delay depends on the dose. In this study, a clear reduction in the proportion of M cells compared to the G2 PCC cells as the dose increased was observed. This observation is similar to those of previous studies performed with low-LET radiation [27,29]. Puig R. et al. observed that—after HPBL irradiation in the dose range 0.5–20 Gy of γ-rays—the proportion of M cells decreased at doses > 1 Gy [29]. These results indicate that, in the context of personalized treatment, it is impossible to identify one single cell stage that gives all of the necessary information. It is important to consider the relevance of findings in studying the effects of the radiotherapy process. Radiation is viewed as an immune-suppressive agent, and most lymphocytes are indeed very sensitive [13]. An understanding of the biochemical and molecular mechanisms in the distinct pathways induced by radiation can facilitate the development of more efficient radiotherapies with beneficial immunological consequences. Moreover, this work suggests a potential direction for further studies, revealing that DNA damage may depend on a specific DNA repair pathway. Consequently, more studies must be performed to compare cell-cycle responses to protons versus conventional photons in other normal and cancer-cell models in order to better understand DNA damage-repair pathways.

## 4. Materials and Methods

### 4.1. Characteristics of the Study Group and Blood Collection

The studied population comprised 10 non-smoking individuals: 5 females (mean age 42.0 ± 4.5 years) and 5 males (mean age 44.0 ± 5.6 years). Detailed information on all examined subjects was obtained via a targeted questionnaire. The donors were healthy at the time of blood sampling. They had no known history of exposure to ionizing radiation, other than that necessary for routine medical diagnosis. The participants signed written informed consent; the study protocol was reviewed and approved by the human bioethical committee of the Regional Medical Board in Krakow (No. 124/KBL/OIL/2013). Phlebotomists collected peripheral blood into vacutainers containing lithium heparin; the samples were then de-identified in the laboratory of The H. Niewodniczański Institute of Nuclear Physics, Polish Academy of Sciences in Kraków, Poland (IFJ PAN).

### 4.2. Proton and X-ray Irradiation and Dosimetry

The proton and X-ray irradiation procedures were previously described in detail [8,9,10,17]. Briefly, the facility used for proton irradiation was the Proteus C-235 isochronous cyclotron, located in the IFJ PAN. After acceleration, the proton beam was delivered to the treatment room by a small field horizontal beamline. The parameters of a fully modulated proton beam with a Spread-Out Bragg Peak (SOBP) and energy of 60 MeV were as follows: 30 mm range, 30 mm modulation (measured in water phantom), and field diameter were collimated to the 40 mm lateral diameter. At the center of the cell container position (i.e., at the depths of 15 mm of the SOBP), the dose-averaged Linear Energy Transfer (LET) was 2.9 keV/µm. Within the sample position in the SOBP, the dose-averaged LET ranged from 2.5 keV/µm to 3.8 keV/µm [8,9,10,17]. A specially designed PMMA-Poly (methyl methacrylate) phantom was placed at the irradiation setup isocentre (in the middle of SOBP) and in the center of the flat beam. Heparinized whole blood samples were irradiated in Eppendorf vials (2 cm long) and set in a phantom at a distance of 93 mm from the final collimator. The chosen doses were as follows: 8.00, 13.64, 15.00, and 20.00 Gy. A pair of non-irradiated samples served as controls (0.0 Gy). The dose range was chosen to mimic the cumulative doses of surrounding healthy tissue when irradiating tumor types (palliative therapy for bones 8.00 Gy, choroidal melanoma fraction dose of 13.64 Gy, 20 Gy dose received by pelvic growth plates).

The proton beam monitoring system and the beam dosimetry have previously been described in detail [8,9,10,17]. The dosimetry of the proton beam was accomplished with the PTW UNIDOS T10001 instrument and the semiflex ion chamber, PTW TM31010. The proton beam intensity was controlled by two transmission PTW ionization chambers, type TM7862, connected to electrometers. Dose measurements were performed in the middle of SOBP using a solid phantom (PMMA). The overall uncertainty of dosimetry was approximately 3%; the precision of dose delivery was better than 0.5%. The average dose rate of the proton beam during irradiation was 0.075 Gy/s. To compare the data with those from photon therapy, blood samples were irradiated with the analogous doses used for the proton beam, using a Phillips X-ray machine operating at the IFJ PAN (model MCN 323, 250 kV, 10 mA, and a dose rate of 0.035 Gy/s). Prior to irradiation of cells, the X-ray dose was measured using the same chamber as for proton beam dosimetry. The vials were held in a polyethylene box. The dimensions of the radiation field were 20 × 20 cm^2^, and the source-to-surface distance was 34.8 cm. All irradiations were performed at room temperature. Immediately after irradiation, the vials with irradiated blood underwent the cytogenetic culturing procedures.

### 4.3. Premature Chromosome Condensation Assay

The PCC test was performed as previously described [17]. To summarize, whole blood (0.5 ml) was added to 4.5 ml of RPMI 1640 culture medium (PAA Laboratories GmbH, Pasching, Austria) and stimulated by adding PHA (10 mg/mL) (Sigma-Aldrich, St. Louis, MO, United States) supplemented with 10% heat-inactivated fetal bovine serum (Gibco, Carlsbad, CA, USA) and antibiotics—100 U/ml penicillin and 100 g/ml streptomycin (Polfa Tarchomin, Warsaw, Poland). The cultures were incubated for 48 h at 37 °C and 5% CO_2_. Exactly 30 minutes before ending the culturing process, calyculin A (50 nM) was added to the culture medium causing the induction of PCC in any phase of the cell cycle (G1, S, G2, M, and A). After 48 hours of cell culture, the cells were harvested according to previously published procedures [17]. The samples were dropped onto an ethanol-washed microscopic slide. Fixed samples were stained with 4% Giemsa in phosphate-buffered water and stored at room temperature until examination. Each measurement point was prepared in triplicate. All slides were coded and blinded to the scorer. Decoding was completed only after the microscopic examination of all slides from the study.

### 4.4. Quantification of Cell Cycle Stages

Each slide was manually scored by one person using the Nikon microscope (Alphaphot-2 YS2) at 400× magnification. The incidences of PCC in cells in the G1, S, G2, M, A phases and nucleated cells were scored according to the criteria developed by Gotoh E. et al. [44]. The cell-cycle stage distribution was calculated as in the formula published by Balakrishnan et al. [45]. Spreads displaying univalent and divalent chromosomes were classified as G1 and G2/M PCC, respectively. PCC in lymphocytes at the S phase had a characteristic mixture of univalent and bivalent chromosomal parts and a “pulverized” appearance [44,45]. G1 PCC are very long, single chromatids; those of G2 are elongated and slender double chromatids; those of the S phase are characterized by their pulverized, fragmented appearance [17,44,45].

### 4.5. Quantification of PCC Fragments and Rings

Cell-finding and image-capturing were performed on a Metafer 4 scanning system equipped with a Zeiss Axio Imager Z2 microscope (MetaSystems^TM^, Altlussheim, Germany). The PCC fragments and rings per cell were manually scored in excess of 46 PCC-condensed chromosomes in 100 G2/M phase cells for each experimental point in an open-access graphic program ImageJ. All excess fragments and rings were presented as a mean value, and the standard deviations (S.D.) were normalized and calculated according to a previously published formula [17].

### 4.6. Statistical Analysis

The Microsoft Office Excel 2013 program was used to perform the data analysis. Figures, as well as Gaussian fitting, were obtained by the OriginPro 2020b (OriginLab, Northampton, MA, USA). The distributions of PCC excess fragments and rings were tested by Papworth’s *u* test. This method utilizes the fact that, for a Poisson distribution, the variance (σ^2^) equals the mean (y). A detailed statistical analysis is given elsewhere [44,46]. The yield (Y) with the error Dispersion index (DI) and *u* values were calculated using the relations described earlier [46]. If the absolute value of *u* is greater than ± 1.96, the over- or under-dispersion is significant. Only a 5% probability exists for the magnitude of *u* to be greater than this value when the distribution is Poisson [44].

## 5. Conclusions

Presented studies demonstrated that high doses of protons induce HPBLs DNA damage in the G2/M phase differently than X-rays. The results indicate a different distri-bution of DNA damage following high doses of irradiation with protons versus photons between donors, types of radiation, and doses. The proliferation index confirms the im-pact of high doses on mitosis and the influence of radiotherapy type on the different HPBLs response.

## Figures and Tables

**Table 1:** The yield ± S.D., dispersion index (DI), and *u*-values (*u*) of excess fragments and rings induced in X-ray and proton-irradiated lymphocytes pooled for each dose from all donors. Values were calculated to test if the distribution of aberrations followed a Poisson.

Excess PCC Fragments						
**X-rays**				**Protons**		
Dose [Gy]	Yield ± S.D.	DI	*u* value	Yield ± S.D.	DI	*u* value
8.00	3.92 ± 0.06	2.58	35.21	4.82 ± 0.07	3.50	55.83
13.64	6.78 ± 0.08	2.31	29.33	8.69 ± 0.09	2.42	31.83
15.00	8.80 ± 0.09	2.24	27.75	9.11 ± 0.10	2.85	41.35
20.00	10.83 ± 0.10	2.33	29.63	13.06 ± 0.11	2.87	41.89
**Rings**						
**X-rays**				**Protons**		
Dose [Gy]	Yield ± S.D.	DI	*u* value	Yield ± S.D.	DI	*u* value
8.00	0.33 ± 0.02	1.12	2.59	0.32 ± 0.02	1.22	4.99
13.64	0.46 ± 0.02	1.44	9.78	0.61 ± 0.02	1.72	16.10
15.00	0.66 ± 0.03	1.72	16.19	0.51 ± 0.02	1.75	16.79
20.00	0.62 ± 0.02	1.35	7.90	0.59 ± 0.02	1.52	11.73

**Table 2:** Percentages of G1, S, G2, M, A phases, and nucleated cells at each dose of X-rays or protons after irradiation with various doses of X-rays or protons. Bold is necessary for showing difference between doses used and results – making it more visible.

X-rays Dose [Gy]	G1	S	G2	M	A	Nucleated	Protons Dose [Gy]	G1	S	G2	M	A	Nucleated
**8.00**	0.05	1.90	4.45	0.05	0.25	93.30	**8.00**	0.00	1.65	2.80	0.05	0.70	94.80
**13.64**	0.05	1.60	2.20	0.00	0.00	96.15	**13.64**	0.00	1.85	1.35	0.00	0.20	96.60
**15.00**	0.00	1.50	2.00	0.00	0.00	96.50	**15.00**	0.05	1.40	1.10	0.00	0.30	97.15
**20.00**	0.00	1.25	1.45	0.00	0.00	97.30	**20.00**	0.10	1.25	0.90	0.00	0.20	97.55

## Data Availability

The data presented in this study are available on request from the corresponding author.

## References

[1] Hu M., Jiang L., Cui X., Zhang J., Yu J. (2018). Proton beam therapy for cancer in the era of precision medicine. J. Hematol. Oncol..

[2] Durante M., Orecchia R., Loeffler J. (2018). Charged-particle therapy in cancer: Clinical uses and future perspectives. Nat. Rev. Clin. Oncol..

[3] Bourhis J., Sozzi W.J., Jorge P.G., Gaide O., Bailat C., Duclos F., Patin D., Ozsahin M., Bochud F., Germond J.-F. (2019). Treatment of a first patient with FLASH-radiotherapy. Radiother. Oncol..

[4] Diffenderfer E.S., Verginadis I.I., Kim M.M., Shoniyozov K., Velalopoulou A., Goia D., Putt M., Hagan S., Avery S., Teo K. (2020). Design, Implementation, and in Vivo Validation of a Novel Proton FLASH Radiation Therapy System. Int. J. Radiat. Oncol. Biol. Phys..

[5] Zhou G. (2020). Mechanisms underlying FLASH radiotherapy, a novel way to enlarge the differential responses to ionizing radiation between normal and tumor tissues. Radiat. Med. Prot..

[6] Sologuren I., Rodríguez-Gallego C., Lara P.C. (2014). Immune effects of high dose radiation treatment: Implications of ionizing radiation on the development of bystander and abscopal effects. Transl. Cancer Res..

[7] Nicosia L., Reverberi C., Agolli L., Marinelli L., de Sanctis V., Valeriani M., Osti M.F. (2019). Long term results of single high dose stereotactic body radiotherapy in the treatment of primary lung tumors. Sci. Rep..

[8] Miszczyk J., Rawojć K., Panek A., Swakoń J., Prasanna P.G., Rydygier M. (2015). Response of human lymphocytes to proton radiation of 60 MeV compared to 250 kV X-rays by the cytokinesis-block micronucleus assay. Radiother. Oncol..

[9] Miszczyk J., Rawojć K., Panek A., Borkowska A., Prasanna P.G., Ahmed M.M., Swakoń J., Gałaś A. (2018). Do protons and X-rays induce cell-killing in human peripheral blood lymphocytes by different mechanisms?. Clin. Trans. Rad. Oncol..

[10] Miszczyk J., Rawojć K. (2020). Effects of culturing technique on human peripheral blood lymphocytes response to proton and X-ray radiation. Int. J. Radiat. Biol..

[11] Buonanno M., Grilj V., Brenner D.J. (2019). Biological effects in normal cells exposed to FLASH dose rate protons. Radiother. Oncol..

[12] Miura T., Nakata A., Kasai K., Nakano M., Abe Y., Tsushima E., Ossetrova N.I., Yoshida M.A., Blakely W.F. (2014). A novel parameter, cell-cycle progression index, for radiation dose absorbed estimation in the premature chromosome condensation assay. Radiat. Prot. Dosim..

[13] Schaue D., McBride W.H. (2012). T lymphocytes and normal tissue responses to radiation. Front. Oncol..

[14] Pike L.R., Bang A., Mahal B.A., Taylor A., Krishnan M., Spektor A., Cagney D.N., Aizer A.A., Alexander B.M., Rahma O. (2019). The Impact of Radiation Therapy on Lymphocyte Count and Survival in Metastatic Cancer Patients Receiving PD-1 Immune Checkpoint Inhibitors. Int. J. Radiat. Oncol. Biol. Phys..

[15] Pujol-Canadell M., Perrier J.R., Cunha L., Shuryak I., Harken A., Garty G., Brenner D.J. (2020). Cytogenetically-based biodosimetry after high doses of radiation. PLoS ONE.

[16] Ravi M., Nivedita K., Pai G.M. (2013). Chromatin condensation dynamics and implications of induced premature chromosome condensation. Biochimie.

[17] Rawojć K., Miszczyk J., Możdżeń A., Swakoń J., Sowa-Staszczak A. (2018). Evaluation of the premature chromosome condensation scoring protocol after proton and X-ray irradiation of human peripheral blood lymphocytes at high doses range. Int. J. Radiat. Biol..

[18] Bourhis J., Montay-Gruel P., Gonçalves Jorge P., Moeckli R., Germond J.-F., Vozenin M.-C. (2019). Clinical translation of FLASH radiotherapy: Why and how?. Radiother. Oncol..

[19] Kacprzak J., Kuszewski T., Lankoff A., Lisowska H., Mueller W.-U., Wojcik A. (2013). Validation of the micronucleus assay for biological dosimetry after high dose exposure. Mutat. Res..

[20] Gotoh E., Durante M. (2006). Chromosome condensation outside of mitosis: Mechanisms and new tools. J. Cell. Physiol..

[21] Lamadrid A.I., García O., Delbos M., Voisin P., Roy L. (2007). PCC-ring Induction in Human Lymphocytes Exposed to Gamma and Neutron Irradiation. J. Radiat. Res..

[22] Panek A., Miszczyk J., Swakoń J. (2018). Biological effects and inter-individual variability in peripheral blood lymphocytes of healthy donors exposed to 60 MeV proton radiotherapeutic beam. Int. J. Radiat. Biol..

[23] Vitti E.T., Parsons J.L. (2019). The Radiobiological Effects of Proton Beam Therapy: Impact on DNA Damage and Repair. Cancers.

[24] Nickoloff J.A., Sharma N., Taylor L. (2020). Clustered DNA Double-Strand Breaks: Biological Effects and Relevance to Cancer Radiotherapy. Genes.

[25] Willers H., Allen A., Grosshans D., McMahon S.J., Neubeck C., Wiese C., Vikram B. (2018). Toward A variable RBE for proton beam therapy. Radiother. Oncol..

[26] Oeck S., Szymonowicz K., Wiel G., Krysztofiak A., Lambert J., Koska B., Iliakis G.E., Timmermann B., Jendrossek V. (2018). Relating Linear Energy Transfer to the Formation and Resolution of DNA Repair Foci After Irradiation with Equal Doses of X-ray Photons, Plateau, or Bragg-Peak Protons. Int. J. Mol. Sci..

[27] Kanda R. (1999). Easy biodosimetry for high-dose radiation exposures using drug-induced, prematurely condensed chromosomes. Int. J. Radiat. Biol..

[28] Guilherme R.S., Ayres Meloni V.F., Kim C.A., Pellegrino R., Takeno S.S., Spinner N.B., Conlin L.K., Christofolini D.M., Kulikowski L.D., Melaragno M.I. (2011). Mechanisms of ring chromosome formation, ring instability and clinical consequences. BMC Med. Genet..

[29] Puig R., Barrios L., Pujol M., Caballín M.R., Barquinero J.-F. (2013). Suitability of scoring PCC rings and fragments for dose assessment after high-dose exposures to ionizing radiation. Mutat. Res..

[30] Park B., Yee C., Lee K.-M. (2014). The Effect of Radiation on the Immune Response to Cancers. Int. J. Mol. Sci..

[31] Liauw S.L., Connell P.P., Weichselbaum R.R. (2013). New Paradigms and Future Challenges in Radiation Oncology: An Update of Biological Targets and Technology. Sci. Trans. Med..

[32] Mosse I., Kilchevsky A., Nikolova N., Zhelev N. (2017). Some problems and errors in cytogenetic biodosimetry. Biotech. Biotechnol. Equip..

[33] Puig R., Pujol M., Barrios L., Caballín M.R., Barquinero J.-F. (2016). Analysis of α-particle-induced chromosomal aberrations by chemically-induced PCC. Elaboration of dose-effect curves. Int. J. Radiat. Biol..

[34] Carter R.J., Nickson C.M., Thompson J.M., Kacperek A., Hill M.A., Parsons J.L. (2018). Complex DNA Damage Induced by High Linear Energy Transfer Alpha-Particles and Protons Triggers a Specific Cellular DNA Damage Response. Int. J. Radiat. Oncol. Biol. Phys..

[35] Kowalska A., Nasonova E., Czerski K., Kutsalo P., Pereira W., Krasavin E. (2019). Production and distribution of chromosome aberrations in human lymphocytes by particle beams with different LET. Radiat. Environ. Biophys..

[36] Carante M.P., Ballarini F. (2016). Calculating Variations in Biological Effectiveness for a 62 MeV Proton Beam. Front. Oncol..

[37] Distela L.V.R., Neubauera S., Kellera U., Carl N., Sprung C.N., Sauera R., Grabenbauer G.G. (2006). Individual differences in chromosomal aberrations after in vitro irradiation of cells from healthy individuals, cancer and cancer susceptibility syndrome patients. Radiother. Oncol..

[38] Terzoudi G.I., Hatzi V.I., Donta-Bakoyianni C., Pantelias G.E. (2011). Chromatin dynamics during cell cycle mediate conversion of DNA damage into chromatid breaks and affect formation of chromosomal aberrations: Biological and clinical significance. Mutat. Res..

[39] Miura T., Blakely W.F. (2011). Optimization of calyculin A-induced premature chromosome condensation assay for chromosome aberration studies. Cytom. A.

[40] Heimers A., Brede H.J., Giesen U., Hoffmann W. (2005). Influence of mitotic delay on the results of biological dosimetry for high doses of ionizing radiation. Radiat. Environ. Biophys..

[41] Romero I., García O., Lamadrid A.I., Gregoire E., González J.E., Morales W., Martin C., Barquinero J.-F., Voisin P. (2013). Assessment of simulated high-dose partial-body irradiation by PCC-R assay. J. Radiat. Res..

[42] Rodríguez P., Barquinero J.F., Duran A., Caballín M.R., Ribas M., Barrios L. (2009). Cells bearing chromosome aberrations lacking one telomere are selectively blocked at the G2/M checkpoint. Mutat. Res..

[43] Wang Z.Z., Li W.J., Zhi D.J., Gao Q.X., Qu Y., Wang B.Q. (2009). Prematurely condensed chromosome fragments in human lymphocytes induced by high doses of high-linear-energy-transfer irradiation. Mutat. Res..

[44] Gotoh E., Asakawa Y., Kosaka H. (1995). Inhibition of protein serine/threonine phosphatases directly induces premature chromosome condensation in mammalian somatic cells. Biomed. Res..

[45] Balakrishnan S., Shirsath K., Bhat N., Anjaria K. (2010). Biodosimetry for high dose accidental exposures by drug induced premature chromosome condensation (PCC) assay. Mutat. Res..

[46] Kanda R., Eguchi-Kasai K., Hayata I. (1999). Phosphatase Inhibitors and Premature Chromosome Condensation in Human Peripheral Lymphocytes at Different Cell-Cycle Phases. Somat. Cell Mol. Genet..

